# Development and validation of artificial intelligence-based analysis software to support screening system of cervical intraepithelial neoplasia

**DOI:** 10.1038/s41598-024-51880-4

**Published:** 2024-01-23

**Authors:** Yung-Taek Ouh, Tae Jin Kim, Woong Ju, Sang Wun Kim, Seob Jeon, Soo-Nyung Kim, Kwang Gi Kim, Jae-Kwan Lee

**Affiliations:** 1 Department of Obstetrics and Gynecology, Korea University Ansan Hospital, 123, Jeokgeum-ro, Danwon-gu, Ansan-si, Gyeonggi-do Republic of Korea; 2 Department of Obstetrics and Gynecology, Konkuk University School of Medicine, 120-1, Neungdong-ro, Gwangjin-gu, Seoul, Republic of Korea; 3 Department of Obstetrics and Gynecology, Ewha Womans University Seoul Hospital, 25, Magokdong-ro 2-gil, Gangseo-gu, Seoul, Republic of Korea; 4 Department of Obstetrics and Gynecology, Institute of Women’s Life Medical Science, Yonsei University College of Medicine, 50-1, Yonsei-ro, Seodaemun-gu, Seoul, Republic of Korea; 5 Department of Obstetrics and Gynecology, College of Medicine, Soonchunhyang University Cheonan Hospital, 31, Suncheonhyang 6-gil, Dongnam-gu, Cheonan-si, Chungcheongnam-do Republic of Korea; 6 R&D Center, NTL Medical Institute, Yongin, Republic of Korea; 7 Department of Biomedical Engineering, Gachon University College of Medicine, Gil Medical Center, 24, Namdong-daero 774beon-gil, Namdong-gu, Incheon, Republic of Korea; 8 Department of Obstetrics and Gynecology, Korea University Guro Hospital, 148, Gurodong-ro, Guro-gu, Seoul, Republic of Korea

**Keywords:** Medical imaging, Biotechnology, Cancer, Health care, Optics and photonics

## Abstract

Cervical cancer, the fourth most common cancer among women worldwide, often proves fatal and stems from precursor lesions caused by high-risk human papillomavirus (HR-HPV) infection. Accurate and early diagnosis is crucial for effective treatment. Current screening methods, such as the Pap test, liquid-based cytology (LBC), visual inspection with acetic acid (VIA), and HPV DNA testing, have limitations, requiring confirmation through colposcopy. This study introduces CerviCARE AI, an artificial intelligence (AI) analysis software, to address colposcopy challenges. It automatically analyzes Tele-cervicography images, distinguishing between low-grade and high-grade lesions. In a multicenter retrospective study, CerviCARE AI achieved a remarkable sensitivity of 98% for high-risk groups (P2, P3, HSIL or higher, CIN2 or higher) and a specificity of 95.5%. These findings underscore CerviCARE AI's potential as a valuable diagnostic tool for highly accurate identification of cervical precancerous lesions. While further prospective research is needed to validate its clinical utility, this AI system holds promise for improving cervical cancer screening and lessening the burden of this deadly disease.

## Introduction

Cervical cancer remains the fourth most common cancer among women worldwide^1–3^. Cervical cancer is a well-known cause of death for women worldwide and develops from precursor lesions known as cervical intraepithelial neoplasia (CIN)^4,5^. It also develops through persistent infection with high-risk human papillomavirus (HR-HPV)^3^. The terms low-grade squamous intraepithelial lesion (LSIL) and high-grade squamous intraepithelial lesion (HSIL) are presently used to describe the histological classification of cervical dysplasia^6,7^. This disease grading is used as a basis for patient management and subsequent treatment, and LSIL and HSIL, respectively, refer to CIN1 and CIN2 or CIN3 subgroups.

Currently, the World Health Organization (WHO) recommends three distinct screening tests: the traditional Pap test with liquid-based cytology (LBC), and visual inspection with acetic acid (VIA)^8^. HPV DNA testing for high-risk HPV types is also recommended. Colposcopic guided biopsy is currently often utilized to diagnose cervical cancer. According to the American Society for Colposcopy and Cervical Pathology (ASCCP) recommendations, those with positive results from cytology or an HPV test were referred for Colposcopy^8^. Patients who were identified as high risk or undetermined by the first two procedures require additional testing and management under the guidance of colposcopy^9^. Clinical experience of colposcopists is a major factor in their ability to reliably identify the characteristics of white epithelial acetate, which is a requirement for colposcopic diagnosis. The absence of skilled inspectors and the substantial workload of screening provide significant obstacles in sets with limited medical services^10^. In order to address the drawbacks of colposcopy, Dr. Stafl from the Medical College of Wisconsin in the United States developed the cervicography system in 1981, which applies the principles of colposcopy to screening^11^. Additionally, NTL Healthcare Co., Ltd. in Korea developed the tele-cervicography system in 2003, a web-based cervicography system that is utilized for cervical cancer screening.

Recently, medical technology has advanced significantly because of artificial intelligence (AI) using machine learning, enabling automated disease detection based on medical image identification^12,13^. As a result, machine learning has been quickly incorporated into the fields of radiology, cardiology, gastrointestinal, and even reproductive medicine. Colposcopic imaging has previously introduced machine learning, however there is currently insufficient data to fully evaluate its specificity and sensitivity^14–17^. The integration of deep learning with digital colposcopy has the potential to enhance automated image classification^18^. However, it remains dependent on the availability of skilled professionals and access to colposcopes, which are often lacking in rural regions of low-income countries. In these areas, utilizing smartphones to capture cervical images and transmit them to colposcopes has been considered as a valuable diagnostic approach, although a recent systematic review revealed suboptimal sensitivity and specificity^19^. In response to these challenges, the scientific community has been actively working on the development of AI-based tools for histological or imaging diagnosis^20^. This represents a promising alternative that could potentially address the aforementioned limitations.

This study used AI-based analysis software (CerviCARE AI) to automatically analyze tele-cervicography images and distinguish between low-grade and high-grade lesions. The purpose of this study was to analyze the sensitivity and specificity of CerviCARE AI as a software used in diagnosing cervical high-grade lesions.

## Material and methods

### Study patients and design

This study is multicenter, blinded, single-arm, retrospective pivotal clinical trial. The clinical validation of the analysis using CerviCARE AI was determined by using the confirmed values of the Independent Evaluation Committee as the reference standard.

Women aged 19 years or older with cervical histologic or cytologic findings were eligible. All patients underwent tele-cervicography and had a tele-cervicography image^21^, and an images with a favorable response to acetic acid application were selected. Images with blood, mucous, cotton ball obstruction, technical defects, myoma, polyp, nabothian cyst, intrauterine device were excluded^22^. We also excluded images with poor quality that compromised the interpretation, as determined by an experienced colposcopist. All methods were performed in accordance with the relevant guidelines and regulations.

### Reference standard

The reference standard was identified and referred to the chairperson of the Independent Review Committee by the Clinical Investigator (CI) who verify the images and histology (or cytology) results assigned an identification code according to the operating procedures of the Independent Review Committee (IRC). The IRC is responsible for establishing reference standards, which are objective read sets against which CerviCARE can evaluate for this study. The chairperson of the independent evaluation committee should verify whether the histology (or cytology) results and images are inconsistent or not. If there is disagreement among three or more members, one member designated as the chairperson of the independent evaluation committee would further review the discrepant images. The verification results should be finalized if three or more people agree. However, if the opinions of less than three members were different, a multilateral meeting of independent evaluators was held to finalize the verification results. The discrepant images would be excluded, confirmed as reference standards, and recorded and archived.

### Primary validation

For the performance test, 400 images were collected from the specimen testing laboratory. The collected images were managed and evaluated by the university hospital, which is the sponsoring institution of the clinical trial. The performance of the model was collected in a retrospective study, starting from April 23, 2022, in reverse chronological order and sequentially. The images were classified into Negative, Atypical, or Positive through the cervicography. For cases with a Positive in cervicography, histologic evaluation was performed. For cases with Negative or Atypical results in cervicography test, Cytology was performed.

By analyzing and evaluating the sensitivity and specificity of CerviCARE AI against the reference standard, we determined that the target sensitivity and specificity for clinical significance in high-risk groups (P2, P3, HSIL or higher, CIN2 or higher) were set at 90% or higher (the guidelines of the Korean Ministry of Food and Drug Safety, MFDS for in-vitro diagnostic medical device), respectively. In addition, the lower limits of the cutoff sensitivity and specificity were determined to be clinically significant at 80% or above, respectively.

### Secondary validation

The sensitivity and specificity of all test groups were analyzed and evaluated by analyzing the results of CerviCARE AI against the reference standard. The positive and negative predictive values were also analyzed.

### Preliminary data

The preliminary study collected 33,531 cervix images. Dr. Cervicam®, a cervical enlargement imaging equipment by NTL Healthcare Co., Ltd., is used for cervical imaging^23,24^. Cervical images are classified into Negative1 (N1), Negative2 (N2), and Atypical (A) for normal, and Positive1 (P0), Positive1 (P1), Positive2 (P2), and Positive3 (P3) for lesions. In the context of colposcopy findings, the positive 0–4 scale is a grading system used to classify and describe the severity of abnormalities observed during a colposcopic examination.

- Negative 1 (N1): has no lesion and components of the transformation zone are visible.
- Negative 2 (N2): has no lesion and components of the transformation zone are not visible.
- Positive 0 (P0) or NILM (Negative for Intraepithelial Lesion or Malignancy): This grade indicates that no abnormal or suspicious findings were observed during the colposcopy. In other words, the examination did not reveal any evidence of intraepithelial lesions or malignancy.
- Positive 1 (P1) or LSIL (Low-Grade Squamous Intraepithelial Lesion): P1 is interpreted as the presence of low-grade squamous intraepithelial lesions. This grade suggests mild abnormalities in the examined tissue. These findings are often associated with human papillomavirus (HPV) infection and represent a relatively low level of concern.
- Positive 2 (P2) or HSIL (High-Grade Squamous Intraepithelial Lesion): P2 indicates the presence of high-grade squamous intraepithelial lesions. This grade suggests more significant and severe abnormalities in the examined tissue. HSIL findings are of greater clinical importance and may be associated with a higher risk of progression to precancerous or cancerous conditions.
- Positive 3 (P3) or Cancer: P3 is used to denote the presence of cancerous cells or malignancy in the examined tissue. In this case, the colposcopy has identified cancer.
- Positive 4 (P4) or Invasive Cancer: P4 is used when the examination reveals invasive cancer. This means that cancerous cells have penetrated surrounding tissues and may be at a more advanced stage. The diagnosis of invasive cancer carries significant clinical implications for treatment planning and prognosis.

### Cervical region detection learning

**Figure 1:**
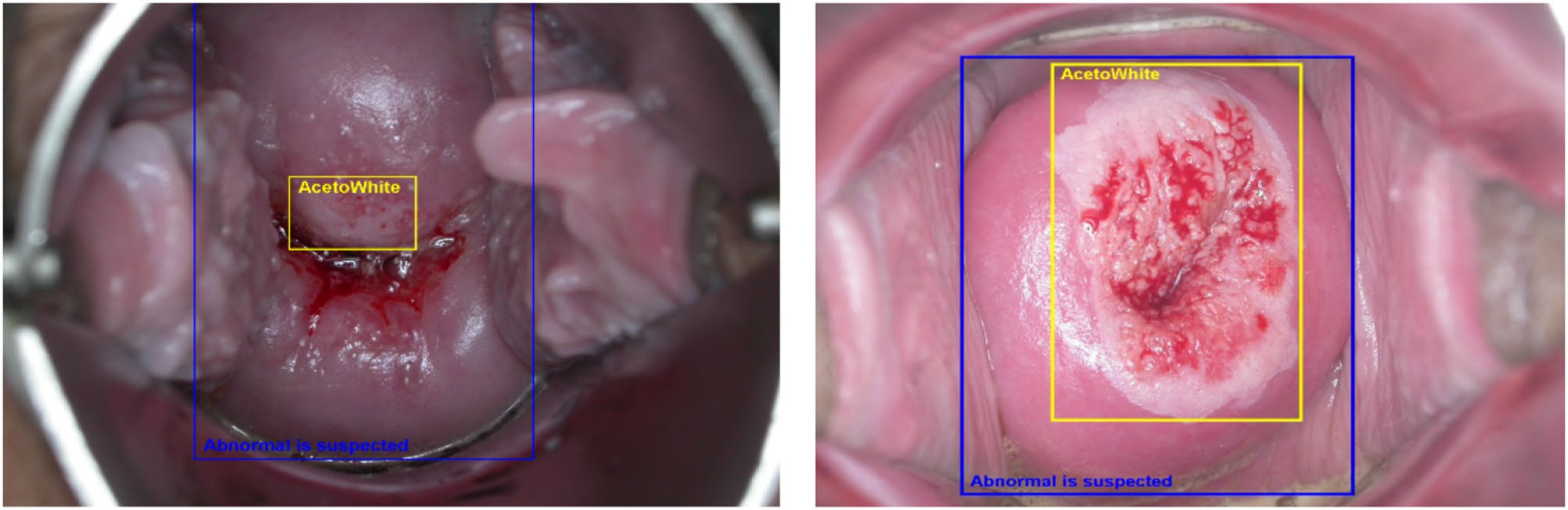
Cervical region detection example.

**Figure 2:**
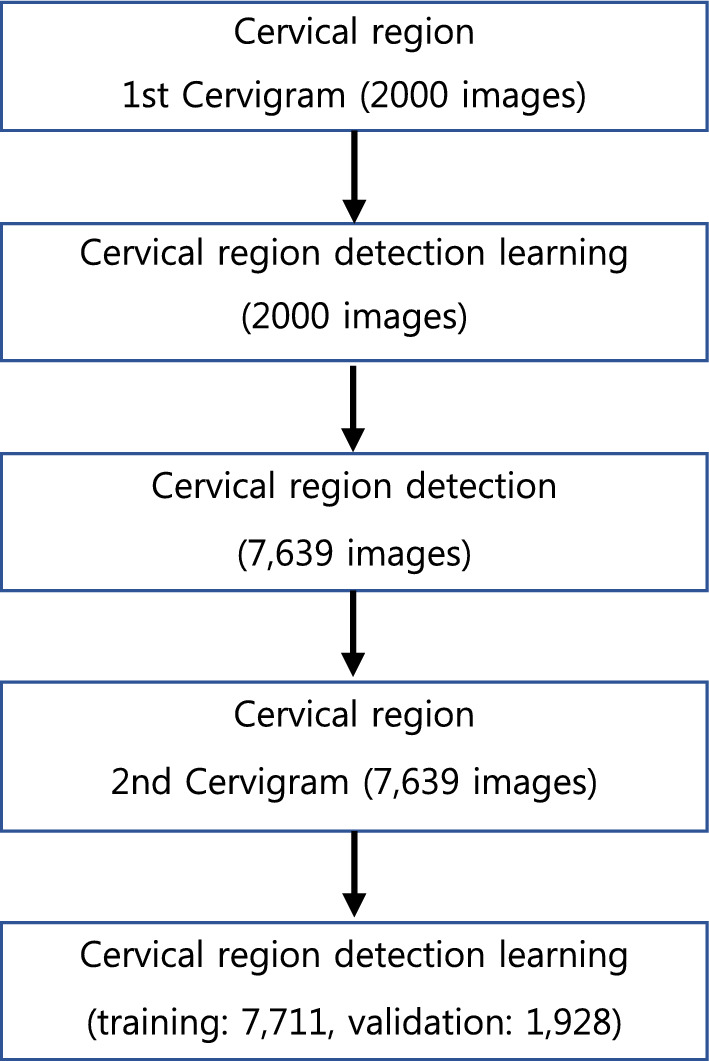
Flowchart of detection learning.

A researcher trained by a specialist on a total of 9639 cervical enlarged photographs (Cervigram™) annotated the cervical area in the form of a box using the Image J program, and all completed data were examined by a specialist (Fig. 1). Using the RetinaNet architecture specialized for detection, 7711 images were used as training set and 1928 images were used as validation set. The ratio of the training set to the validation set was 8:2 (Fig. 2).

### Cervical image preprocessing

Using the detection model, only the cervical region is extracted in the form of box for the new Cervigram™ data and processed using image sharpening^25^, Contrast Limited Adaptive Histogram Equalization (CLAHE)^26^, and color uniformization techniques to optimize classification.

Images sharpening is an image processing technique that improves edge contrast and noise by filtering high-pass values of images and adding them to the original. Accordingly, the image becomes clear as a whole, and the boundaries between the elements in the image are better revealed^25^.

Histogram equalization is an image processing technique that converts pixel values concentrated in a specific range to be evenly distributed. When pixel values are redistributed by applying histogram equalization, the contrast of the entire image is improved^27^. In addition, the contrast between the useful pixel value and the surrounding pixel value increases, making it easier to recognize the difference, and a clearer and better image quality image can be obtained.

CLAHE is an image processing technique that divides an image into square tiles of a certain size and improves the contrast for each tile so that the image becomes uniform overall. Unlike HE, the contrast is limited to produce less noise, and the transformed image is similar to the real image^26^.

### Cervical cancer classification learning

**Figure 3:**
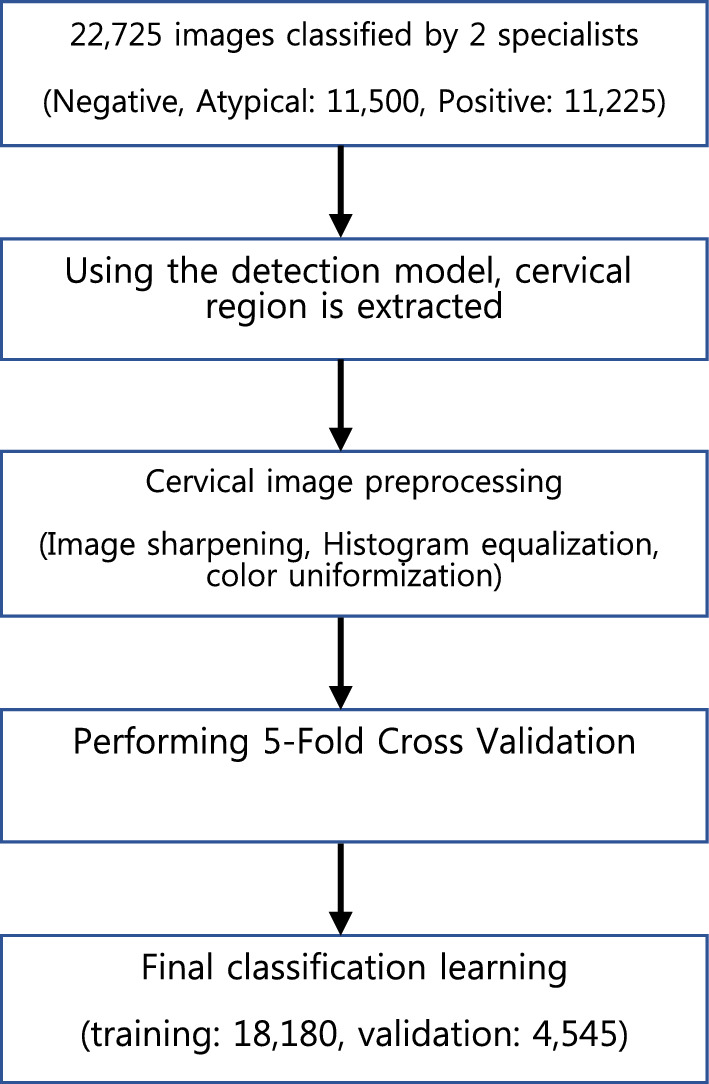
Flowchart of classification learning.

There are 22,725 images to be used for classification learning, all of which are in the form of a box that includes only the cervical region through the image preprocessing (Fig. 3). Of the total data, 11,500 images are included in the Negative and 11,225 images are included in the Positive class (Table 1).

**Table 1:** Composition of images used in the learning.

Data	Negative		Positive				Total
Class	Normal	Atypical	P0	P1	P2	P3	
Original	9001	2499	1200	2409	6000	1616	22,725

The total number of images is 22,725, which is divided into five subsets as evenly as possible. The following results were obtained by performing 5-folds cross validation (Table 2). Each of the 5 folds consisted of an equal number of images, with a 1:1 ratio of positive to negative samples within each fold. As a result of performing 5-folds cross validation, the performance of the model was verified to be appropriate, 18,180 images are used as training set, and 4545 images are used as validation set. Preliminary detection model separately selected and applied cervical image proprocessing techniques in advance. Classification was learned into Negative and Positive classes using a classification-specific ResNet-50 architecture. Due to the fast execution speed of ResNet-50, we selected this algorithm.

**Table 2:** Result of 5-Fold Cross Validation.

5-Fold cross validation	TP	FP	FN	TN	Sensitivity, %	Specificity, %	Accuracy, %
CV 0	1636	325	609	1973	72.9	85.9	79.4
CV 1	1986	510	257	1790	88.5	77.8	83.1
CV 2	1974	274	272	2026	87.9	88.1	88.0
CV 3	1970	268	276	2032	87.7	88.4	88.0
CV 4	1780	306	465	1996	79.3	86.7	83.0
Average	–				83.3	85.4	84.3

TP, true positive; FP, false positive; FN, false negative; TN, true negative.

### Classification Model Performance Assessment

For the performance test, 400 images that were not used for learning were prepared. This set includes 160 negative images, 40 atypical images, and 200 positive (P0, P1, P2, and P3) images. The positive images consisted of images in which both the cervicography test results and either the Cytology test results or Pathology test results were positive (Table 3).

**Table 3:** Composition of test images.

	N	A	P0	P1	P2	P3	Total
Test Set	160	40	19	81	85	15	400
TP, TN	146	31	16	66	81	15	355
FP, FN	14	9	3	15	4	0	45

N, normal; A, atypical; TP, true positive; FP, false positive; FN, false negative; TN, true negative.

The test results are as follows:

- Accuracy : 355/400 = 0.8875
- Specificity : 177/200 = 0.885
- Sensitivity : 178/200 = 0.89
- Sensitivity of P2, P3 images : 96/100 = 0.96

### Cervical cancer inference process

**Figure 4:**
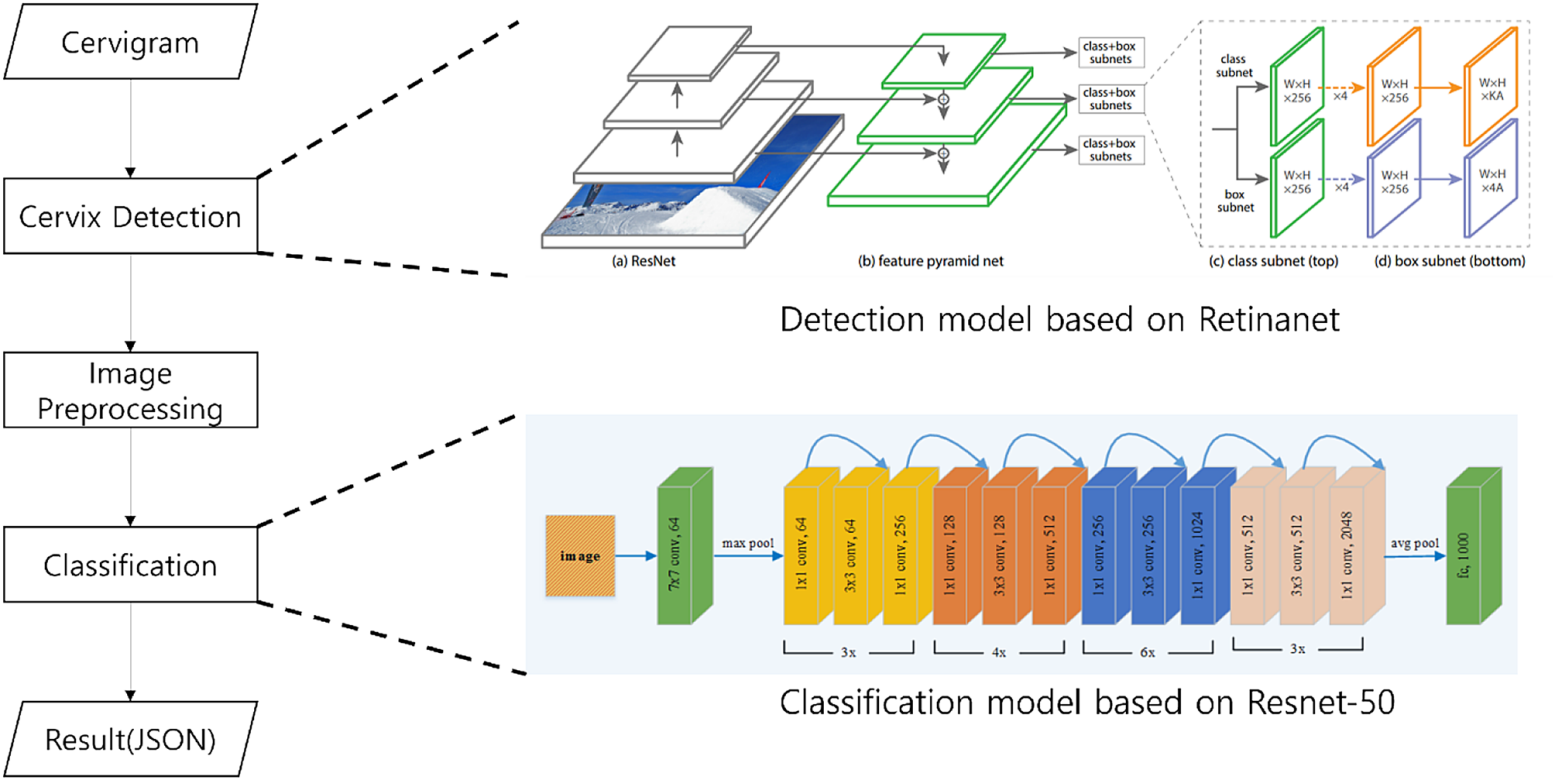
Inference process of CerviCARE AI.

The image to be classified is input in the form of jpg, and the cervical region is detected and extracted using a detection model. The extracted cervical region images go through a preprocessing process, are applied to the classification model, and are finally classified into a Negative or Positive class (Fig. 4).

### Statistical analysis

Sensitivity and Wald 95% confidence intervals are presented using instrument measurements obtained from high-risk patients (P2, P3, HSIL or higher, CIN2 or higher) among standard data positives. In addition, Clopper-Pearson 95% confidence intervals were used. If the lower limit of the 95% confidence interval was higher than 80%, it was considered clinically significant.

### Ethical approval

This study was approved by the Institutional Review Board (IRB) of Korea University Guro Hospital prior to data extraction (IRB No. 2021GR0555). The requirement for informed consent was waived by the IRB due to its retrospective nature.

## Results

### Study participants

**Figure 5:**
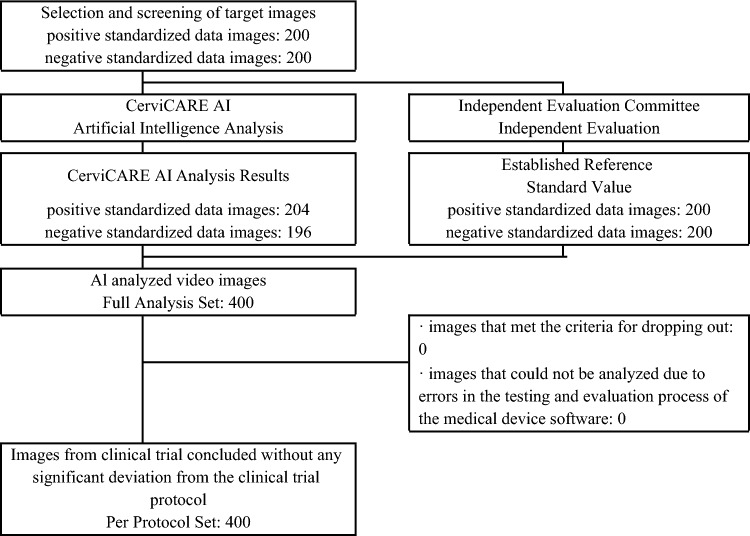
Flowchart of the study.

For this validation study, a total of 400 images were selected as sample data, including 200 negative standardized data images and 200 positive standardized data images from the validated tele-cervicography images. In the Full Analysis Set (FAS), a total of 400 negative and positive images were included in the test set of the medical device software were analyzed. In the Per Protocol Set (PPS), the same number of 400 images as in the FAS were analyzed, as there were no images that met the criteria for dropping out and no images that could not be analyzed due to errors in the testing and evaluation process of the medical device software (Fig. 5).

### Primary validation

Analyzing a total of 400 slides with CerviCARE AI, the sensitivity against high-risk groups (P2, P3, HSIL or higher, CIN2 or higher) is 98.0% with a Wald 95% confidence interval of 0.953 to 1.000. The sensitivity was 98.0% and the lower limit of the target sensitivity was 95.3%, which is considered clinically significant (Table 4).

**Table 4:** The diagnostic performance for detecting high-risk groups (P2 or P3).

	Software (CerviCARE AI)	
	Positive	Negative
Reference Standard (Independent Evaluation Committee Reading + Histology, Cytology)		
Positive (P2 or P3)	98	2
Negative	9	191
Sensitivity (95% CI)*	98.0% (0.953–1.000)	
Specificity (95% CI)*	95.5% (0.926–0.984)	

*95% Wald confidence interval.

### Secondary validation

For the overall study population, CerviCARE AI achieved a sensitivity of 97.5% and a specificity of 95.5%, with a Wald 95% confidence interval of 0.953 to 0.997. The positive predictive value was 95.6% with a Wald 95% confidence interval of 0.928 to 0.984, and the negative predictive value was 97.4% with a Wald 95% confidence interval of 0.952 to 0.997. Sensitivity for P0/P1 was 97.0%, specificity for P2/P3 was 98.0%, and specificity for negative lesions was 95.5% (Tables 5 and 6).

**Table 5:** The diagnostic accuracy for all lesions.

Reference Standard (Independent Evaluation Committee Reading + Histology, Cytology)	Software (CerviCARE AI)		
	Positive	Negative	Total
Positive	195	5	200
Negative	9	191	200
Total	204	196	400
Sensitivity (95% CI)*	97.5% (0.953–0.997)		
Specificity (95% CI)*	95.5% (0.926–0.983)		
PPV^†^ (95% CI)*	95.6% (0.928–0.984)		
NPV^‡^ (95% CI)*	97.4% (0.952–0.997)		

*95% Wald confidence interval.

^†^Positive predictive value.

^‡^Negative predictive value.

**Table 6:** The diagnostic performance according to the grade of the lesions.

	Software (CerviCARE AI)	
	Positive	Negative
Reference Standard (Independent Evaluation Committee Reading + Histology, Cytology)		
Positive (P0 or P1)	97	3
Positive (P2 or P3)	98	2
Negative	9	191
Sensitivity (P0 or P1)*	0.97	
Sensitivity (P2 or P3)*	0.98	
Specificity	0.955	

## Discussion

Improving the accuracy of colposcopy is the most important factor in early detection of cervical cancer. Even for experienced colposcopists, there can be errors in accurate diagnosis through colposcopy^28,29^. The role of cervical cancer screening through CerviCARE AI has become more important recently. Based on this, we developed a cervical cancer diagnosis technology using artificial intelligence and validated its usefulness through 400 patients including 200 normal and 200 abnormal tele-cervicography images. The aim of this study in applying CerviCARE AI is whether it is useful for classification of cervical intraepithelial neoplasia (CIN) and valuable tool for screening.

Compared to a reference standard certified by a panel of experts, CerviCARE AI demonstrated a very high level of sensitivity of 98.0% for high-grade lesions and a specificity of 95.5%. Accuracy for all lesions, including those of negative lesions, was also very high, with sensitivity of 97.5%, specificity of 95.5%, positive predictive value of 95.6%, and negative predictive value of 97.4%. This compares favorably to 82.2% agreement with histologic findings in other systems^30^.

Cervical precancerous lesions are categorized into LSIL and HSIL. HSIL is more likely to progress to cervical cancer and requires biopsy or excision such as conization^31^. Therefore, it is important to detect HSIL in order to prevent it from progressing to cervical cancer. LSIL usually occurs as a change in the cervix due to a transient infection with HPV and often regresses spontaneously^32^. The sensitivity of clinician performed colposcopy to detect HSIL is not known to be higher than 80% according to previous studies^18,33^. CerviCARE AI, which we used in this study, has a sensitivity and specificity of more than 95.0%, although we used carefully selected images. This positively predicts the clinical feasibility of CerviCARE AI-based colposcopy over traditional colposcopy.

Colposcopy is a technique for inspecting and assessing the cervix in real-time in order to find CIN and invasive cancer. Since decades, there has been debate about the reliability of colposcopy and colposcopy-guided biopsy in identifying high-grade CIN and cervical cancer. It was suggested that the colposcopy be carried out by a skilled and well-educated clinician to decrease erroneous diagnosis and subsequent inappropriate treatment in order to minimize the potential harm caused by the colposcopy and biopsy^34^. Colposcopic examination and biopsy are less reliable, may ignore a substantial percentage of high-grade CIN that is common, and have a false-negative rate that ranges from 13 to 69%^35–37^. However, in actual clinical practice, there was frequently a lack of skilled colposcopists in the countries where cervical cancer was a serious disease burden. Some advised performing a multiple biopsy and randomized biopsy from the quadrants with normal appearance to increase the sensitivity of colposcopy-guided biopsy^38,39^.

Furthermore, the development of a cloud-based AI platform to provide accessible remote healthcare support in resource-poor settings, such as rural villages with a lack of skilled colposcopists and colposcopy services, could address disparities in healthcare. It is already well known that inequalities in cervical cancer screening due to socioeconomic factors have increased^40,41^, and the introduction of AI to cervical cancer screening is expected to play a large role in solving this problem. Thus, it can be expected to meet the demand for standardized cervical cancer screening and diagnosis methods, reduce the diagnostic capacity gap between tertiary and primary hospitals, raise the standard of screening programs, and facilitate collaboration to expand screening coverage globally.

Several previous investigators have demonstrated the feasibility of AI in colposcopy. In 2014, Simoes et al.^42^ demonstrated a diagnostic accuracy of 72.2%, and Miyagi et al.^18^ demonstrated 82.3% accuracy, 80.0% specificity, and 88.8% sensitivity in detecting HSIL. Comparing our results with those of previous published data, we found that CerviCARE AI exhibits competitive or superior performance. For instance, Miyagi et al.^18^ developed a CNN-based AI classifier for LISIL/HSIL classification in colposcopy images, with a sensitivity of 80.0% for HSIL diagnosis. Our system's sensitivity surpasses this value, indicating its ability to accurately detect high-risk lesions. Additionally, our AI's specificity of 95.5% compares favorably with the specificity values reported in other studies, such as the ResNet model by Yuan et al.^43^, which had a specificity of 82.62%. While the C-RCNN algorithm proposed by Yue et al.^44^ achieved exceptional specificity and sensitivity, our system's results remain competitive in the context of cervical lesion classification. Furthermore, while the proposed method achieved a respectable classification accuracy of 86.3%, a sensitivity of 84.1%, and a specificity of 89.8% in a limited dataset^45^, our AI system significantly outperforms with offering superior diagnostic accuracy for the identification of cervical precancerous lesions. This stark contrast in sensitivity and specificity highlights CerviCARE AI's potential to greatly improve early detection and reduce disparities in cervical cancer screening, especially in regions with limited access to experienced colposcopists, underscoring its clinical significance and utility.

There are several limitations to this study. First, although CerviCARE AI showed satisfactory high sensitivity, the study was retrospective and only included images that were validated by experts. In this regard, its usefulness in real-world clinical practice should be evaluated prospectively, especially in terms of accuracy and cost-effectiveness. In addition, there are various clinical variables such as polyps and condyloma in real world, but we have excluded all of them. To further verify the AI system’s predictive performance, a prospective research including randomized controlled trial is necessary. However, it is noteworthy that several studies focusing on AI applications in colposcopy or cervicography, including our own, predominantly demonstrate technical feasibility rather than extensive clinical utility. This observation underscores the necessity for prospective clinical trials to rigorously evaluate the potential for actual clinical adoption of these AI technologies. Nevertheless, based on its consistency in grading colposcopy impressions and ordering biopsies, we have identified the potential to introduce CerviCARE AI into colposcopy clinics as an accurate and complementary diagnostic tool for colposcopists during colposcopy procedures.

## Conclusion

CerviCARE AI accurately predicted expert-verified images of tele-cervicography with very favorable sensitivity and specificity. Despite existing constraints in the clinical deployment of CerviCARE, its value as an supplemental resource has been verified. Further research is needed to determine whether it can be applied in clinical practice.

## Data Availability

The datasets generated during and/or analyzed during the current study are available from the corresponding author on reasonable request.
